# Preliminary study on a novel dedicated plate for iliac fractures in dogs

**DOI:** 10.1371/journal.pone.0269313

**Published:** 2022-08-26

**Authors:** Tryssia S. M. Moi, Bruno W. Minto, Ana P. Macedo, Dayvid V. F. Lucena, Caio A. S. Malta, Luis G. G. G. Dias

**Affiliations:** 1 Department of Veterinary Clinic and Surgery, Faculty of Agrarian and Veterinary Sciences, São Paulo State University (UNESP), Jaboticabal, São Paulo, Brazil; 2 Department of Dentistry, Faculty of Dentistry, University of São Paulo (USP), Ribeirão Preto, Brazil; USP FZEA: Universidade de Sao Paulo Faculdade de Zootecnia e Engenharia de Alimentos, BRAZIL

## Abstract

**Introduction:**

The aim of this study was to describe and evaluate a novel plate designed specifically for the canine ilium using finite element methods. The cranial portion of the plate had an elliptical shape and contained nine screw holes. The caudal portion of the plate was triangular with three screw holes. Four and three screws were used at the cranial (screw 1 to 4) and caudal (screw 5 to 7) segment of the plate. Finite element models of the plate and canine pelvis were created. A load of 300 N was applied on the femur-hip joint contact area. Values of Von Mises Stress on the plate, screws and the maximum and minimum main stresses in the bone were used to capture the mechanical factors in this study.

**Results:**

The novel implant had a plate stress of 51.9 megapascals (MPa) with higher stress in the dorsal part of the plate. Screws 2 and 4 showed similar stress values of 17.3 MPa. Screws 1 and 3 were the most loaded (51.9 MPa and 75 MPa, respectively). Screws 5, 6, and 7 showed similar dissipation and stress values (21.55 MPa). There was traction force in the dorsal region of the pubis and compression in the ventral part, with dissipation and values of 15.4 MPa and 23.9 MPa, respectively, acquiring balance between them.

**Conclusion:**

The novel plate is potentially applicable and specifically suitable for iliac fractures in dogs. The screws distribution modeled proved itself strategical since the simulated stresses were evenly distributed throughout the implant construct.

## Introduction

Fractures of the pelvis account for approximately 25% of all reported fractures in polytraumatized dogs [1–3]. They occur usually after high energy trauma such as motor vehicle induced injuries, or high-level falls and 18 to 46% of these pelvic fractures usually compromise the ilium. Iliac fractures are generally obliquely displaced and are often accompanied by concurrent pubic fractures and sacroiliac luxation [4–6].

Surgical management of iliac fractures usually involves open reduction and lateral contoured-plate application fixed by screws [7, 8]. Other surgical techniques include dorsal or ventral ilium plating, interfragmentary compression achieved by K-wires or lag screws, reduction by screw-wire-polymethylmethacrylate (SWP) composite and/or external skeletal fixation device [2, 4, 9]. Despite such assortment of repair methods available, none of them is properly fit to ensure anatomical shape reduction. This may explain the considerable 21% complication rate, composed mostly of implant-related setbacks [6, 7, 10, 11].

Screw loosening is a common cause of implant-related complication and can subsequently result in poor fracture reduction, narrowing of the pelvic canal, medial acetabular displacement and might scale up to non-unions [2, 4, 12]. Some authors have suggested the poor-quality bone stock in the cranial ilium to be the cause of the high frequency of screw loosening [8, 10]. Several plating techniques have been employed for the repair of iliac fractures such as veterinary cuttable plates, String-of-Pearls™ plates, T plates, Tibial plateau levelling osteotomy locking-compression plates (TPLO), Locking Compression Plates (LCP) and reconstruction plates, in an attempt to optimize better quality bone purchase [6–13].

Custom-made implants developed based on specific anatomical requirements have decreased complication rates in veterinary surgery, noticeable on the prolific recent advances and expanding plate generations suitable for proximal tibial osteotomies to treat cruciate disease in companion animals like TPLO systems, Tibial Tuberosity Advancement (TTA) options, Cora Based Levelling Osteotomy (CBLO) techniques, to cite a few [14]. Even further, patient-specific customized implants developed for people made considerable stiffness improvements compared to prior designs when resisting tension and torsional shear stresses with decreased risk of implant-related failure such as screw loosening [13, 15].

The purpose of this study was to design an anatomically shaped bone plate proper for canine iliac fracture and characterize its properties by finite element modelling. We hypothesized that the novel implant model would properly fit the ilium surface in a strategic anatomical shape with optimal screw distribution. Thus, we aimed at creating a custom-iliac-shaped specific implant suitable for the challenging surgical repair of fractured dogs.

## Materials and methods

### Ethics committee

This study employed canine cadavers and was conducted in accordance with the institutional animal ethics policies and guidelines. It has been approved by the local Ethics Committee on the State University of São Paulo (UNESP)—Jaboticabal / SP, under the protocol (n° 07283/19).

### Geometric details and implant design

**Fig 1:**
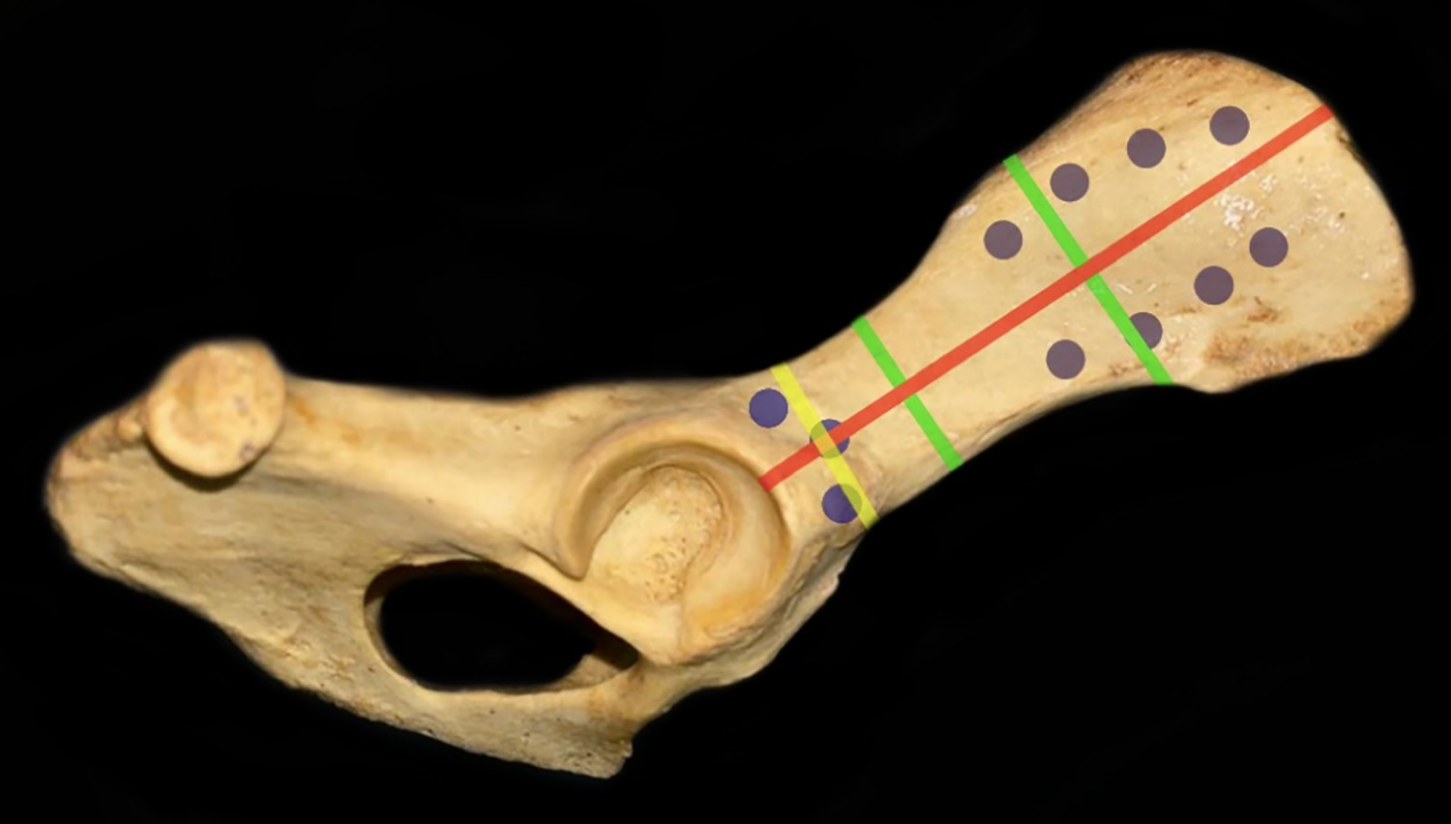
Schematic representation of the landmarks used for pelvic assessments performed on dog cadavers to ensure fitness and determine optimal shape dimensions of the novel canine iliac plate.

Five dog cadavers that weighed between 25 and 30 kg were included in this study. We collected and measured their pelvic girdle (Table 1) to record their pelvic shape and extrapolate the average geometrical dimensions of the novel canine iliac plate (CIP). We aimed at ensuring a universal shape that would fit all the included cadaveric pelvic models. We recorded measures of their pelvic bones in four different landmarks: iliac wing width (value 1), iliac body width (value 2), the width of the region cranial to the acetabulum (value 3) and the length from the cranial acetabular ridge to the iliac crest (value 4). These landmarks are displayed on Fig 1.

**Table 1:** Measures of cadaveric pelvic dog bones.

	Value 1	Value 2	Value 3	Value 4	Breed	Weight	Age
Pelvis 1	3.6 cm	2.4 cm	2.6 cm	10.2 cm	Boxer	27.3 kg	4 years
Pelvis 2	3.3 cm	2.2 cm	2.4 cm	9.9 cm	Bull Terrier	26.2 kg	3 years
Pelvis 3	3.3 cm	2.2 cm	2.7 cm	9.5 cm	Chow Chow	28.1 kg	4 years
Pelvis 4	3.5 cm	2.3 cm	2.7 cm	9.9 cm	Mixed Breed	25.8 kg	2 years
Pelvis 5	3.7 cm	2.3 cm	2.2 cm	9.5 cm	Golden Retriever	28.9 kg	3 years

cm: centimeter

We performed a throughout literature review to expand our understanding of the canine pelvis and the main risk factors that may impair iliac osteosyntheses. We mapped the pelvic region to set the landmarks according to bone stock quality, allowing better bone purchase by the screws, especially in the cranial aspect of the ilium. We also identified the anatomical landmarks considering possible plate designs and suitable shapes that ought to ensure balanced stress distribution and, therefore, avoid overloading situations that could loosen the screws.

**Fig 2:**
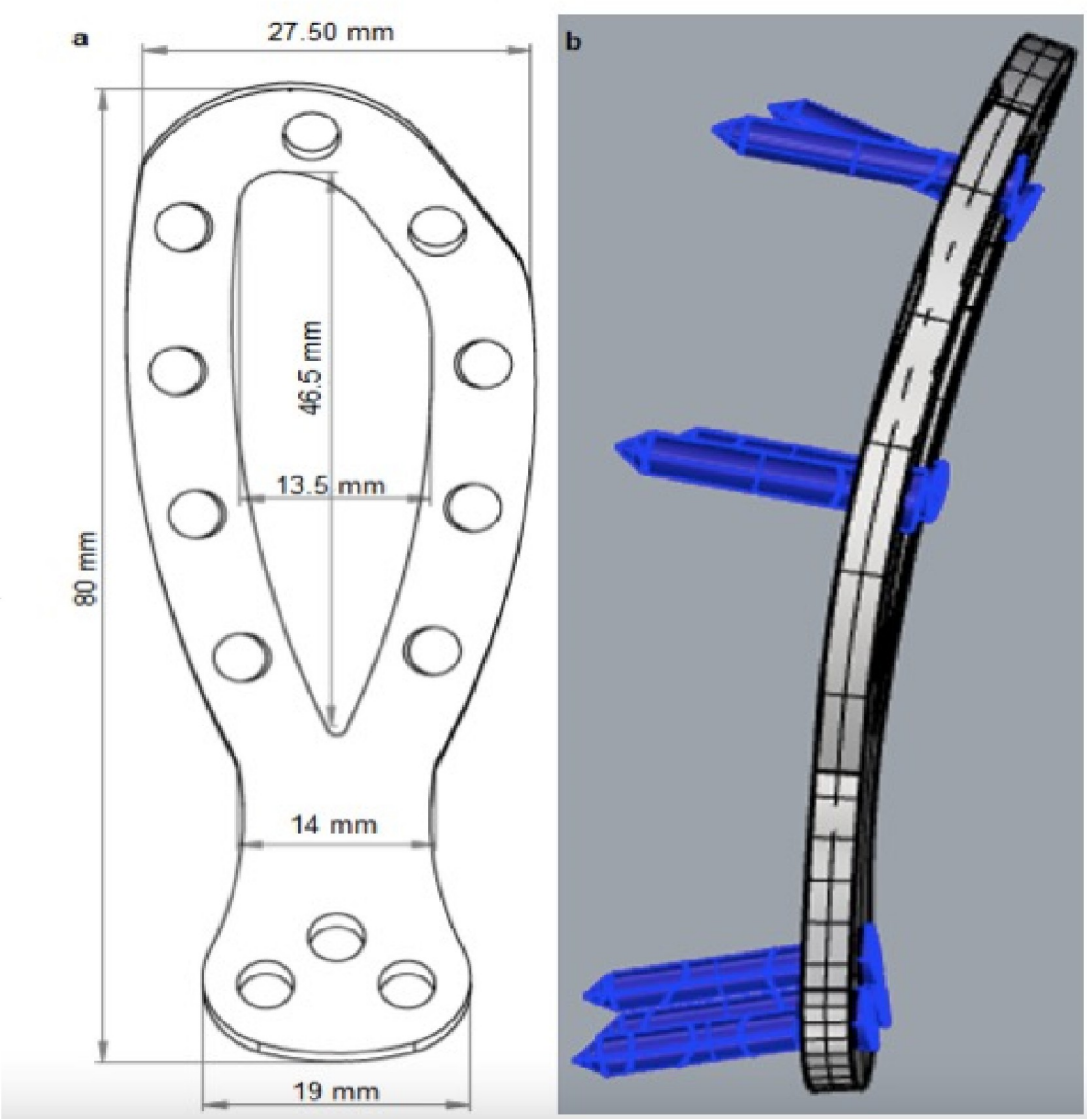
Schematic representation of the new model plate with measurements (a) and pre-direction of the screws (b).

The novel plate model was then created in an elliptical shape measuring 80 mm in length and 27.5mm and 19mm in width in the cranial and caudal regions, respectively; the transition area from the cranial to the caudal region was 14mm wide and 3mm thick. The central area of the ellipse was hollow and measured 13.5mm in width and 46.5mm in length (Fig 2A), allowing the design to follow the shape of the wing and the body of the ilium and be more versatile.

The cranial part of the plate, consisting of two branches, formed a semi-ellipse with four holes in each branch and a hole in the cranial curvature. The caudal part had a triangular shape with three holes. The holes of the cranial portion were 6° divergent, directed toward the edge of the ilium, due to the higher bone density found in this region. Conversely, the caudal holes in the plate were directed caudally at an angle of 12° for fixation into better bone stock (Fig 2b). The plate and screw set were equivalent to a 3.5-mm system with a pre-defined contour angle of 27°, with 20-mm-long screws and a 2.8-mm core.

A pelvis without fracture of a young dog (25 kg, mixed-breed) that had died from causes unrelated to the study was produced using computed tomography data (Shimadzu SCT -7800 CT, Kyoto, Japan). All images were processed in a “*stl”* file in cross section by Invesallius software to identify the outline of various hard tissues (cortical and spongy bone).

**Fig 3:**
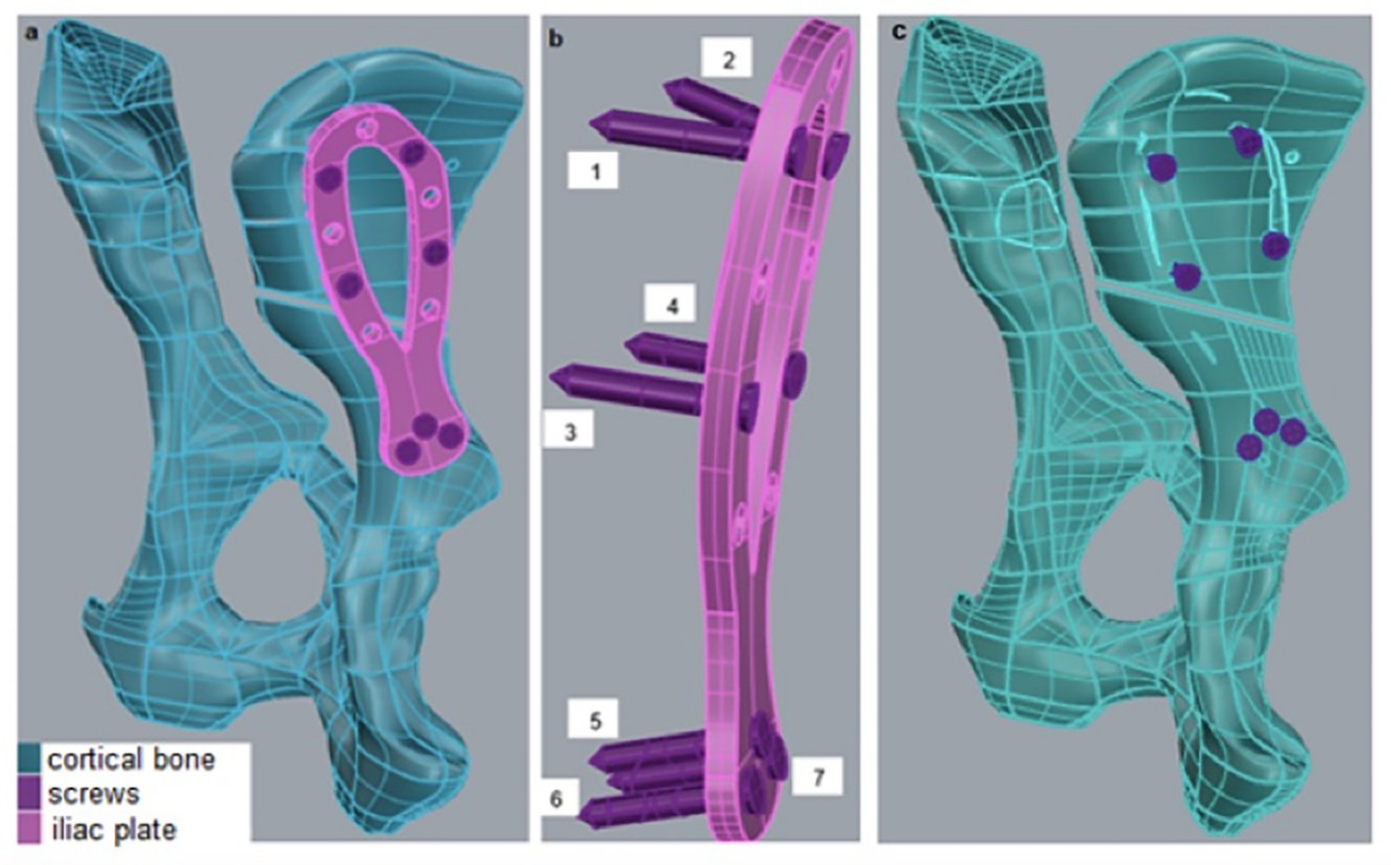
Lateral plate mounted on the osteotomized pelvis in tridimensional illustration (a). Detail of the screws inserted (b). Bone model illustration without the bone plate to denote the anatomical position of the osteotomy gap in relation to the body of the ilium (c).

The virtual development of the plate was performed using computer aided design according to the measures previously mentioned. The file was exported in a step format to Rhinoceros 6.0 (McNeil, Assoc USA). In Rhinoceros, the pelvis design was developed with an existing gap of 2 mm in the right hemipelvis, as an oblique fracture in the iliac body, in addition to the setting plate and the screws to the pelvis. The screws used for fixing the plate to the pelvis were distributed in such a way that four were located in the cranial portion (two in each semi-ellipse), with the other three located in the caudal portion of the plate, as shown in Fig 3. The screws were fixed using the freeze contact.

### FE analysis

We exported the model images to SimLab software (2018 version). Then we developed tridimensional pelvic mesh based on four-node isoparametric tetrahedral finite element models. We also added the novel plate design held by the bone screws. We used an average 0.6 mm element size to differentiate cortical from cancellous bone, whereas we used 0.8 mm-sized plate elements and 0.9 mm-sized screw elements for the computed model.

The CIP system was considered as being made of 316L stainless-steel so we could assess the implant’s strength and define its rigidity as a fixation method. We based our expected knowledge of the material’s properties on previous data found on the literature. In such sense, we considered cortical bone to have modulus of elasticity (E) of 17000 MegaPascals (MPa) and a 0.25 Poisson’s ratio (v), while cancellous bone was thought to have E = 776 MPa and v = 0.3. Values of 316 L stainless-steel alloy of E = 200,000 MPa and v = 0.3 were used for modeling the plate and screws [8, 16–18].

The contact between the surfaces were defined as slides used to detect mechanical contact between the plate and the pelvis. The mechanical contact between the screws and the osteotomized pelvis was defined via a glue contact between the matching nodes in the contact bodies. The proposed plate will work with locking screws and so the contact between the plate and screw has been considered glued. The same boundary conditions were applied in the FE model to evaluate the different stiffness values.

A 300 N load was applied to all FE models [11]. This net force was composed of loads on the x, y and z axes, and the resulting load vector was directed to the ventromedial region. The constraining function that would be exerted by the sacrum and the lumbar spine on a real patient was mimicked by the nodes connecting the pelvic bone to the vertebral spine. We proceeded to a static analysis then.

### Data analysis

The von Mises equivalent principal stresses provide a convenient representation of the resulting stresses on the given plate, as well as on the screws and the maximum and minimum main stresses on the bone, that is why we sought to evaluated it. We quantitatively analyzed the stresses generated on the external and internal surfaces of the implant’s body and its respective components using HyperView^™^ GER’s software. We took the results in account to assess and record the largest areas values of stress concentration.

## Results

### Stress distribution on screws

**Fig 4:**
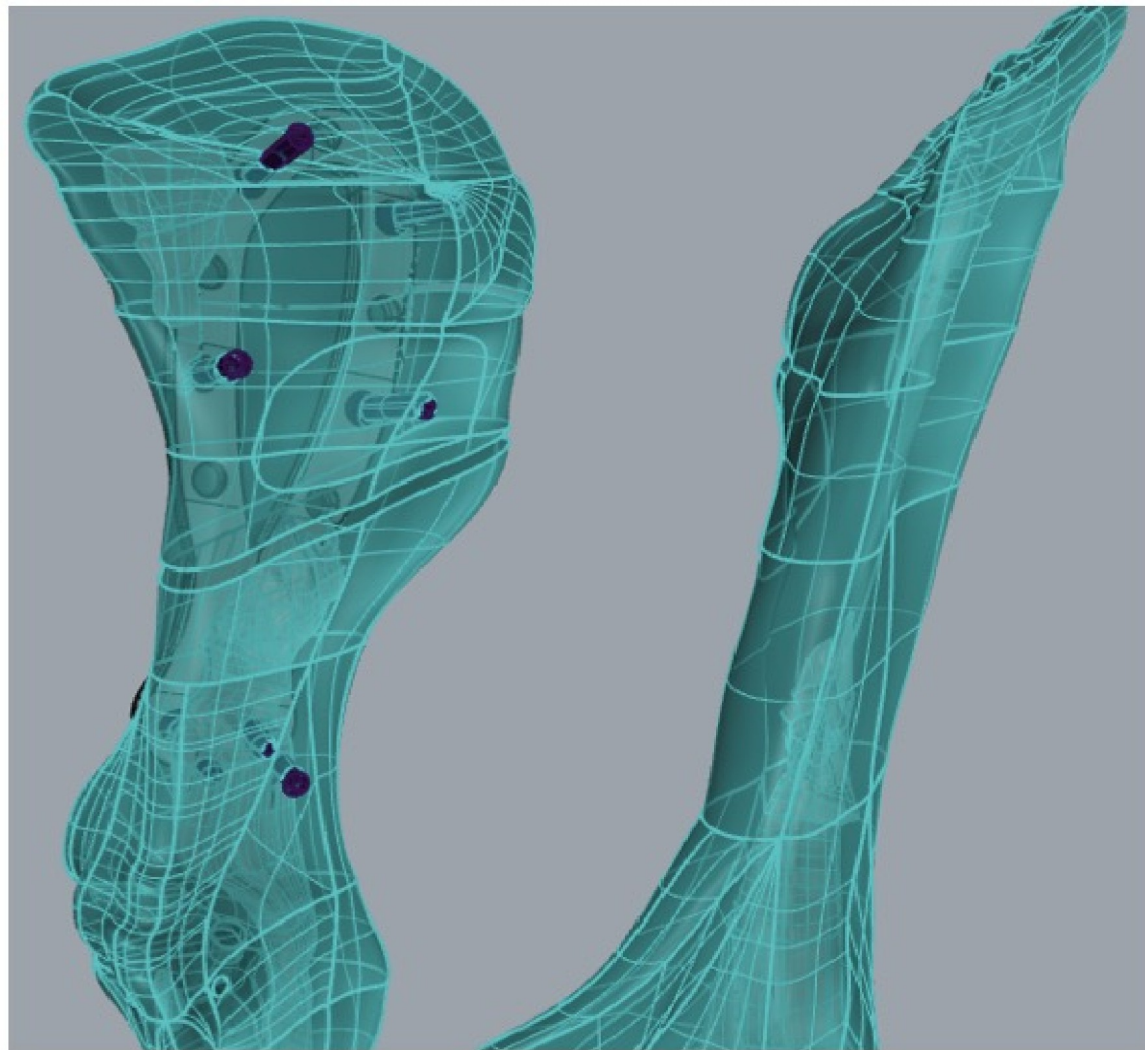
Medial view of the screws inserted in the cranial and caudal segments of the ilium.

The caudal screws, with 12° of caudal inclination, allowed suitable bone stock screw purchase (Fig 4). The cranial screws, in being 6° divergent with cranial inclination, could be placed in parts of the ilium with significant good bone purchase and, additionally, multiples screws could be used in the same segment.

**Fig 5:**
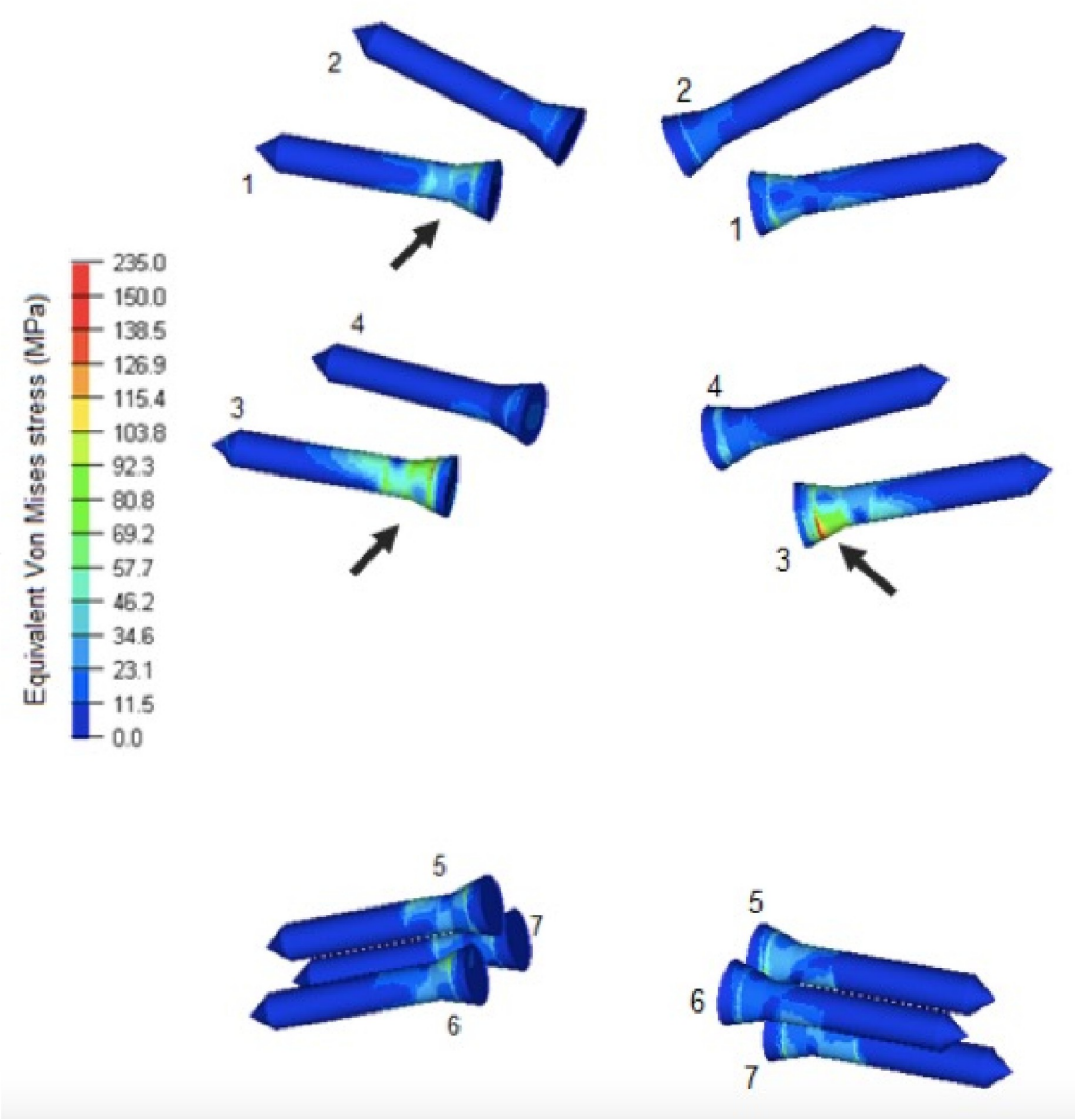
Von Mises stresses in megapascals obtained from studying the inserted seven inserted screws in the dorsal and ventral view.

Screws 2 and 4 were the least loaded and withstanded similar stress of 17.3 MPa. Screws 1 and 3 underwent the highest stresses, reaching 51.9 MPa and 75 MPa, respectively. There was similar stress and dissipation in screws 5, 6 and 7, with a mean value of 40.4 MPa (Fig 5). Screws seem to have had all the same point of tension concentration in the region of the screw body, close to the head of the screw (Table 2).

**Table 2:** Values of Von Mises stress in the screws.

	Screw von Mises stresses on screws	Traction stress values in the orifice of screws in cortical bone	Compression stress values in the orifice of screws in cortical bone
Screw 1	51.9 MPa	Null	Null
Screw 2	17.3 MPa	Null	Null
Screw 3	75 MPa	Null	23.9 MPa
Screw 4	17.3 MPa	Null	Null
Screw 5	40.4 MPa	21.55 MPa	19.25 MPa
Screw 6	40.4 MPa	21.55 MPa	19.25 MPa
Screw 7	40.4 MPa	21.55 MPa	19.25 MPa

MPa: megapascal

### Implant stress distribution

**Fig 6:**
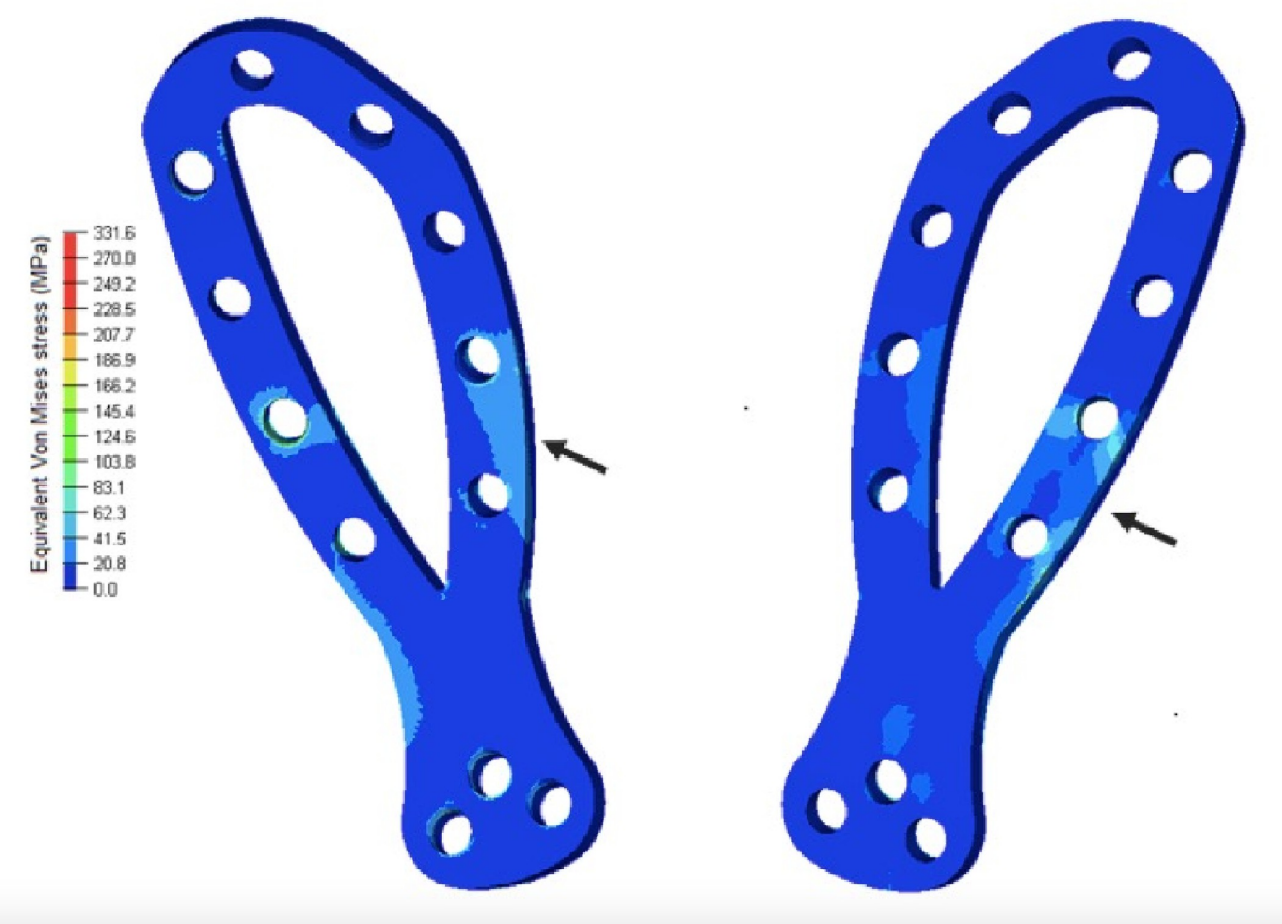
Von Mises stress in megapascals in the internal and external region for the novel iliac plate.

The most loaded region of the cranial part, mainly dorsal region, of the CIP was close to the osteotomy gap (indicated by the black arrow in Fig 6) in each semi-ellipse, with tension values of 51.9 MPa. In the caudal region, there was proper dissipation of forces between the plate and the screws. The intrinsically predefined angulation adequately created the potential possibility of proper positioning.

### Distribution of stress in cortical bone

The insertion sites of screws 3, 5, 6, and 7, and the dorsal region of the pubis experiences traction forces in the pelvis. Compression loading occurred in the ventral region of the pubis and in the insertion sites of screws 3, 5, 6, and 7.

The values for traction of the regions were 15.4 MPa for the pubis and screws 5, 6, and 7, and 21.55 MPa for screw 3. The compression values for the pubis and screw hole 3 were the same (23.9 MPa). The insertion sites of screws 5, 6, and 7 showed a mean value of 19.25 MPa (Table 2).

## Discussion

In view of the broad theoretical and clinical foundations demonstrating the benefits of dedicated surgical implants in human medicine and dentistry [19], veterinary medicine must also move in this direction, which has already occurred for several procedures [12, 14, 15]. There is a lack of implants available for the surgical repair of iliac fractures; the present research bridges this gap with an implant developed specifically for the ilium, with promising benefits from its design and observed results.

During the development of the canine iliac plate (CIP), we sought to overcome the most challenging problems of lateral fixation of iliac fractures, in particular poor bone stock in the cranial segment for screw fixation [6] and reconstruction of the anatomical curvature of the ilium [5, 13, 16, 20]. Achieving better bone purchase was maximized with divergent angle configurations of the holes in the cranial segment of the plate, allowing the screws to be positioned in a thicker peripheric region of the ilium, more dorsally or ventrally. This potentially increased the implant’s capacity to distribute load and resist forces.

The elliptical shape and multiple divergent holes in the cranial portion of the implant can, potentially, improve fixation to the iliac wing and cranial portion of the body of the ilium, which is usually performed using non-specific implants such as the TPLO plate or double plating fashion [6, 8]. These adaptations have improved the plate’s bone purchase in the cranial segment [6, 10]; however, it may not improve force distribution.

The anatomical pre-contoured 27° angled shape contributed to restore the iliac curvature. In a hypothetical surgical scenario, it could potentially fit the bone surface without further need of plate contouring. This fact, allied with the strategical divergent angles of the screws, met our expectations of the plate’s full potential and desired mechanical behavior of the plate [13].

During the analyses, it was observed that the most loaded screws were one and three. That might be explained by the overloading of the location of the screws—based on mechanical forces acting on the ilium region and the hip joint that are known to be highest loading scenario [19, 21]. The region has endured the highest tension because it is suffering compressive loads on its surface during load applications. The homogeneous and strategical distribution of the screws make the CIP potentially more resistant than straight or adapted plates, which tend to have specific stress concentration. This increases the chance of implant failure, usually represented by fractures in the caudal segment and/or screw loosening [6, 9]. Further studies are necessary to objectively demonstrate mechanical differences between the CIP and other plates.

Screw 3 was the closest to the fracture line and withstanded maximum tension of 80.8 MPa. Considering that a common complication of iliac surgeries is loosening of the cranial screw [2, 10, 20], by being near to the fracture line and not being overloaded—the minimum stress recorded was 87.52 MPa [3, 6], we believe that the novel design has potential to avoid such complications in a hypothetical clinical scenario.

The inverted triangular base shaft (caudal end) of the CIP allowed the positioning of three slightly angled screws, improving implant reach of good quality bone stock just cranially to the acetabulum, optimizing the expected fitness for any given plate on such landmark [14]. Implant application in this region can be challenging depending the size of the fractured fragments and the surgical options available, markedly reduced if one only has conventional long straight plates [4, 13, 14, 22].

Different materials behave differently, and future studies are necessary. Titanium’s modulus of elasticity resembles that of the bone and not so much that of the stainless steel. Steel resists less to cyclic fatigue and tends to plastically deform easier [23].

Summary, the load distribution between the cranial and caudal portions of the CIP can be acknowledge. Based on our results and considering the forces and stress dissipations, we believe that it is possible to predict the elasticity of the plate. Extrapolating to a clinical scenario, we hypothesize that the CIP might provide a mechanical-friendly behavior that would prevent interfragmentary motion.

This study describes the steps to develop a novel bone implant and the finite elements data analysis of the same bone plate customed to fit the canine ilium. The novel plate is potentially applicable and specifically suitable for iliac fractures in dogs. Ours was an *in vitro* study and further comparative and biomechanical studies are necessary to clinically validate our implant.

## Supporting information

S1 File (PDF)
