# Supplementary material for: Preliminary study on a novel dedicated plate for iliac fractures in dogs
Source: PLoS One. 2022 Aug 26;17(8):e0269313. doi: 10.1371/journal.pone.0269313 (PMC9416997; doi:10.1371/journal.pone.0269313)
Supplement: S1 File — (PDF) [file pone.0269313.s001.pdf]

UNIVERSIDADE ESTADUAL PAULISTA
"JÚLIO DE MESQUITA FILHO"
Câmpus de Jaboticabal

CEUA – COMISSÃO DE ÉTICA NO USO DE ANIMAIS

CERTIFICADO

Certificamos que o projeto de pesquisa intitulado **"Desenvolvimento, caracterização e análise por elementos finitos de novo modelo de placa para osteossintese ilíaca em cães"**, protocolo nº 07283/19**,** sob a responsabilidade do Prof. Dr. Bruno Watanabe Minto, que envolve a produção, manutenção e/ou utilização de animais pertencentes ao Filo Chordata, subfilo Vertebrata (exceto o homem), para fins de pesquisa científica (ou ensino) - encontra-se de acordo com os preceitos da lei nº 11.794, de 08 de outubro de 2008, no decreto 6.899, de 15 de julho de 2009, e com as normas editadas pelo Conselho Nacional de Controle de Experimentação Animal (CONCEA), e foi aprovado pela COMISSÃO DE ÉTICA NO USO DE ANIMAIS (CEUA), da FACULDADE DE CIÊNCIAS AGRÁRIAS E VETERINÁRIAS, UNESP - CÂMPUS DE JABOTICABAL-SP, em reunião ordinária de 13 de junho de 2019.

Vigência do Projeto	17/06/2019 a 01/10/2020
Espécie / Linhagem	*Canis familiaris*
Nº de animais	1 animal
Peso / Idade	30 kg / Porte médio
Sexo	Não se aplica
Origem	Será obtido uma pelve canina para confecção do molde de um modelo de placa.

Jaboticabal, 13 de junho de 2019.

Fabiana Pilarski

Prof.ª Dr.ª Fabiana Pilarski
Coordenadora – CEUA

Faculdade de Ciências Agrárias e Veterinárias
Via de Acesso Prof. Paulo Donato Castellane, s/n CEP 14884-900 - Jaboticabal/ SP - Brasil
tel 16 3209 7100 www.fcav.unesp.br
